# Cost-effectiveness of increasing vaccination in high-risk adults aged 18–64 Years: a model-based decision analysis

**DOI:** 10.1186/s12879-018-2967-2

**Published:** 2018-01-25

**Authors:** Angela R. Wateska, Mary Patricia Nowalk, Richard K. Zimmerman, Kenneth J. Smith, Chyongchiou J. Lin

**Affiliations:** 1 Department of Medicine, University of Pittsburgh School of Medicine, 200 Meyran Ave., Suite 200, Pittsburgh, PA 15213 USA; 2 Department of Family Medicine, University of Pittsburgh School of Medicine, Pittsburgh, PA USA

**Keywords:** High-risk adults, Pneumococcal vaccine, Influenza vaccine, Tdap vaccine, Primary care, Adult vaccination

## Abstract

**Background:**

Adults aged 18–64 years with comorbid conditions are at high risk for complications of certain vaccine-preventable diseases, including influenza and pneumococcal disease. The 4 Pillars™ Practice Transformation Program (4 Pillars Program) increases uptake of pneumococcal polysaccharide vaccine, influenza vaccine and tetanus-diphtheria-acellular pertussis vaccine by 5–10% among adults with high-risk medical conditions, but its cost-effectiveness is unknown.

**Methods:**

A decision tree model estimated the cost-effectiveness of implementing the 4 Pillars Program in primary care practices compared to no program for a population of adults 18–64 years of age at high risk of illness complications over a 10 year time horizon. Vaccination rates and intervention costs were derived from a randomized controlled cluster trial in diverse practices in 2 U.S. cities. One-way and probabilistic sensitivity analyses were conducted.

**Results:**

From a third-party payer perspective, which considers direct medical costs, the 4 Pillars Program cost $28,301 per quality-adjusted life year gained; from a societal perspective, which adds direct nonmedical and indirect costs, the program was cost saving and more effective than no intervention. Cost effectiveness results favoring the program were robust in sensitivity analyses. From a public health standpoint, the model predicted that the intervention reduced influenza cases by 1.4%, with smaller decreases in pertussis and pneumococcal disease cases.

**Conclusion:**

The 4 Pillars Practice Transformation Program is an economically reasonable, and perhaps cost saving, strategy for protecting the health of adults aged < 65 years with high-risk medical conditions

**Electronic supplementary material:**

The online version of this article (10.1186/s12879-018-2967-2) contains supplementary material, which is available to authorized users.

## Background

Adults aged 18–64 years with immunocompromising and other chronic medical conditions are becoming a sizable proportion of the U.S. population; of U.S. adults aged 50–64 years, 30.6% had a least one of these conditions [1]. These individuals are at high risk for complications of certain vaccine-preventable diseases, including influenza and pneumococcal disease [2]. Yet, vaccination rates among all adults aged 19–64 years are low overall, with influenza vaccination coverage from 31.5–47.7% and tetanus vaccination 62.6–64.7% among all 19–64 year olds, and pneumococcal vaccination in only 20.3% of high-risk individuals in this age group [3]. Few studies have examined attempts to overcome barriers to vaccine uptake in 19–64 year olds.

A recent exception is the 4 Pillars™ Practice Transformation Program (4 Pillars Program) which has increased vaccination rates among varied populations in diverse medical practices [4–8]. The program, developed through CDC support and owned and licensed by the University of Pittsburgh, consists of medical practice-based tools to improve: 1) the convenience of vaccination services, 2) communication with patients about the importance of immunization and the availability of vaccines, 3) office systems to facilitate immunization, and 4) motivation through an office immunization champion who monitors progress and encourages adherence to vaccination-promoting office practices. Although the 4 Pillars Program (4pillarstoolkit.pitt.edu) requires a commitment to long-term practice systems changes, it has been shown to be cost-effective among adults aged 65 years and older [9], but its cost-effectiveness in high-risk non-elderly adults is unknown.

Using changes in vaccination rates as a result of the 4 Pillars Program, where trial-based vaccination rates in high-risk adults aged 18–64 improved by 5–10%, and cost estimates for implementing the program, we examined the cost-effectiveness of the 4 Pillars Program for improving vaccination rates among adults 18–64 years of age with immunocompromising and other chronic medical conditions that confer a high risk of vaccine-preventable disease. This analysis was motivated, in part, by the modest program-related improvement in vaccination rates carrying with it a relatively high burden of investment.

## Methods

**Fig. 1:**
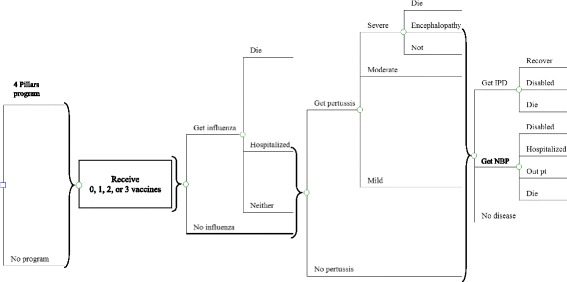
Decision Tree diagram. At the square decision node, identical hypothetical cohorts of high-risk 18–64 year olds could receive the implementation program or not. Nodes to the right of brackets are connected to all branches to the left of brackets. At each circular chance node, potions of cohorts could receive vaccines, become ill with influenza, pertussis, and/or pneumococcal disease, with or without complications, based on the 10-year probability of those events. Disease probabilities were based on vaccines received and vaccine effectiveness. IPD = invasive pneumococcal disease; NBP = non-bacteremic pneumococcal pneumonia

Using a decision tree model (Fig. 1), constructed in TreeAge Pro 2017 (TreeAge Software, Williamstown MA), the cost-effectiveness of implementing the 4 Pillars Program in primary care practices was compared to no program in identical hypothetical cohorts of high-risk adults 18–64 years of age. High-risk adults were defined, using CDC definitions, as those with one or more comorbid or immunocompromising conditions, with comorbid conditions including one or more of the following: chronic heart, lung, or liver diseases, alcoholism, diabetes mellitus, or individuals who smoke cigarettes; immunocompromising conditions were HIV disease, hematologic malignancies, dialysis, nephrotic syndrome, organ or bone marrow transplant, sickle cell disease, immune deficiency, or current immunosuppressive therapy. Pneumococcal polysaccharide vaccine (PPSV), influenza vaccine, and tetanus-diphtheria-acellular pertussis (Tdap) vaccination rates and intervention costs from a randomized controlled cluster trial implementing the 4 Pillars program in two U.S. cities were used. The cohort of high-risk 18–64 year-olds not receiving the intervention was assigned baseline vaccination rates for high-risk 18–64 year olds from the trial, while an identical cohort receiving the intervention had absolute increases in vaccination uptake rates for this age and risk group from the end of the two-year trial. Ranges of vaccine coverage in both intervention and non-intervention cohorts came from uptake rates in different trial sites. Because our unit of analysis was the cohort and overall vaccine protection within the cohort, we assumed that the probability of receiving one vaccine was independent of receiving the others and, based on these probabilities, portions of the cohort could potentially receive one, two, or three vaccines, or none at all. Despite knowing that persons receiving one vaccine are more likely to receive another, we assumed this independence because there is no cross protection from one vaccine to another. Thus, protection afforded by any one vaccine is solely determined by the proportion of the population receiving that vaccine. Based on current guidelines regarding pneumococcal immunization, we assumed that immunocompromised persons receive both PPSV and the 13-valent pneumococcal conjugate vaccine (PCV13), while the remaining high-risk persons received PPSV only.

**Fig. 2:**
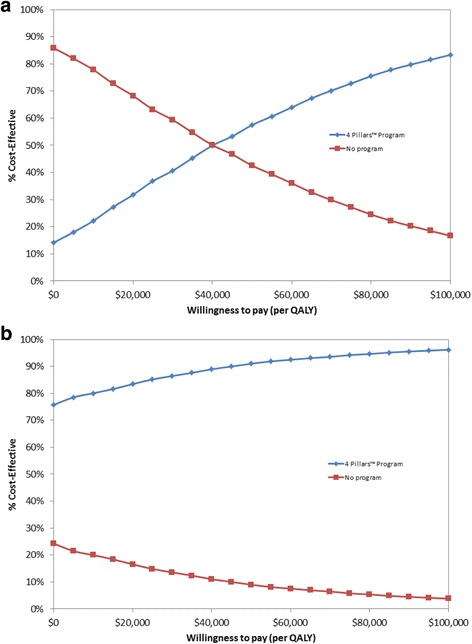
Probabilistic sensitivity analysis. Cost-effectiveness acceptability curves showing the likelihood each strategy will be favored over ranges of willingness to pay (or acceptability) thresholds when all parameters are varied simultaneously over distributions: **a** third-party payer perspective, **b** societal perspective

Table 1 lists model parameter base case values and their corresponding ranges examined in sensitivity analyses. Each parameter was varied individually in 1-way sensitivity analysis over the ranges shown in Table 1. All parameters were also simultaneously varied over their distributions 5000 times in a probabilistic sensitivity analysis, with results depicted in Fig. 2 as acceptability curves. As the original study did not collect illness rate data, illness attack rates for unvaccinated high-risk individuals 18–64 years of age were derived from the medical literature; whereas illness risk for vaccinated individuals was calculated as each illness’s attack rate in the unvaccinated multiplied by 1 minus vaccine effectiveness. Age-specific estimates of vaccine effectiveness for influenza and Tdap were obtained from the medical literature. For pneumococcal vaccine, age- and comorbidity-specific Delphi expert panel estimates [10] of yearly effectiveness – PPSV against invasive pneumococcal disease (IPD), PCV13 against IPD, and PCV13 against non-bacteremic pneumococcal pneumonia (NBP) – were each averaged over a 10-year period, with overall effectiveness calculated as illness-specific vaccine effectiveness multiplied by the vaccine-specific pneumococcal illness serotype prevalence to calculate illness-specific vaccine effectiveness against all serotypes, adjusted for recent pneumococcal vaccine trial results [11]. In the analysis, we assume that PPSV is ineffective against NBP, based on Delphi panel estimates [10], which could bias against interventions to improve vaccination rates. Over the model 10-year time horizon, program-related improvement in vaccination rates was assumed to be constant. However, these improvements were varied widely in sensitivity analyses to test this assumption, and to determine what levels of improvement would be necessary for the program to be favored.

**Table 1:** Model parameter values for high-risk adults aged 18–64 years

Parameter	Base case	Range	Source
*Probabilities*	*%*	*%*	
Vaccination probability with no program			
Influenza	52.1	26.4–85.7	4 Pillars™
Tdap	37.9	4.2–85.7	4 Pillars™
Pneumococcal vaccines	43.4	16.7–61.9	4 Pillars™
Absolute increase in vaccine uptake with program			
Influenza	4.7	0–15.2	4 Pillars™
Tdap	11.5	0–27.3	4 Pillars™
Pneumococcal vaccines	12.3	4.1–28.6	4 Pillars™
Vaccine effectiveness			
Influenza	59.0	20–67	[28]
Tdap (10 year average)	24.5	0–95	[29]
Pneumococcal vaccines (10 year average ^a^)			Calculated [10, 11]
PPSV alone (pts with comorbid conditions) Against IPD	46.5	22–72	
Against NBP	0	–	
PPSV and PCV13 (immunocompromised pts) Against IPD	36.3	19–56	
Against NBP	25.8	14–40	
Pneumococcal illness serotype prevalence			
PCV13 serotypes	30.7	6.8–63	[16]
PPSV serotypes	67.6	51–82	[16]
Relative likelihood of immunocompromised given high-risk	10.7%	5–15%	[1]
Probability of illness without vaccinations (yearly)			
Influenza	6.6	3.2–10	[12]
Pertussis	0.202	0.101–0.303	[15]
IPD (pts with comorbid conditions)	0.012	0.006–0.018	[13]
IPD (immunocompromised pts)	0.074	0.037–0.111	[13]
NBP (pts with comorbid conditions)	1.44	0.72–2.16	[13]
NBP (immunocompromised pts)	9.05	4.5–13.58	[13]
Relative likelihood of outpatient treatment (vs. inpatient)	90.07	76–98	[13]
IPD disability	6.02	4–8	[13]
IPD mortality	15.9	13.8–35.2	[13]
NBP disability	3	2–4	Estimate
NBP mortality	6.3	5.3–14.3	[13]
Case-hospitalization, influenza	1.93	0.65–3.21	[12]
Case-mortality, influenza	0.134	0.04–.224	[12]
Outpatient influenza	62.5	38.9–86.1	[12]
Pertussis severity relative likelihood			
Mild	11	5–17	[14]
Relative likelihood of treatment (vs. no treatment)	37.2	20–55	[14]
Moderate	86	75–90	[14]
Severe (hospitalized)	3	0–6	[14]
Encephalopathy, given severe	1.43	0–3	[14]
Mortality, given severe	0.86	0–2	[14]
*Costs (base year 2015)*	*US$*	*US$*	
Vaccines			
Influenza	10.69	6.64–32.75	[30]
Tdap	37.55	20.18–42.61	[30]
PPSV	78.90	26.60–130	[30]
PCV13	159.60	96.1–220	[30]
Vaccine administration, per vaccine	25.08	20–30	[31]
Implementation program, per eligible person	1.78	0.70–2.26	4 Pillars™
Mild pertussis, when treated			
Third-party payer perspective	305	153–457	[17]
Societal perspective	882	441–1323	[17]
Moderate pertussis			
Third-party payer perspective	424	212–636	[17]
Societal perspective	1001	501–1502	[17]
Severe pertussis			
Third-party payer perspective	7850	3925–11,775	[14]
Societal perspective	8261	4130–12,391	[14]
Influenza (outpatient)	944	472–1416	[12]
Hospitalized influenza	53,212	26,606–79,818	[12]
Pneumococcal disease			
Invasive pneumococcal disease	30,745	15,373–46,118	[13]
Non-bacteremic pneumococcal pneumonia (hospitalized)	17,466	8733–26,199	[13]
Non-bacteremic pneumococcal pneumonia (outpatient)	571	286–857	[13]
Disability	32,987	16,494–49,481	[13]
Cost of death	153,085	76,543–229,628	[12]
Cost of lost productivity	671,226	335,613–1,006,839	[12]
Cost of lost day of productivity	187	158–223	[12]
*Utilities*			
Influenza			
Outpatient	0.558	0.3–0.8	[17]
Hospitalized	0.2	0.1–0.4	Estimate
Pertussis			
Mild	0.9	0.8–0.99	[14]
Moderate	0.85	0.75–0.95	[14]
Severe	0.81	0.6–0.9	[14]
Encephalopathy	0.2	0–0.4	[14]
Non-bacteremic pneumococcal pneumonia			
Inpatient	0.2	0–0.5	Estimate [32]
Outpatient	0.9	0.7–1	Estimate
Invasive pneumococcal disease	0.2	0–0.5	[32]
Disability post pneumococcal disease	0.4	0.2–0.6	Estimate [33]
*Disutilities (quality adjusted life years lost)*	*QALY*	*QALY*	
Illness death (discounted)	10.25	5–15	[18]
*Durations (days lost due to illness)* *Illness duration (days)*			
Influenza	*Days*	*Days*	
Outpatient	4	1–8	[12]
Hospitalized	24	15–35	[12]
Pertussis	87	68–107	[14]
Non-bacteremic pneumococcal pneumonia			
Inpatient	27	18–38	[34]
Outpatient	18	11–26	[34]
Invasive pneumococcal disease	27	18–38	[34]

*Tdap* Tetanus, diphtheria, pertussis vaccine, *IPD* Invasive pneumococcal disease, *NBP* Non-bacteremic pneumococcal pneumonia, *PCV13* 13-valent pneumococcal conjugate vaccine, *PPSV* Pneumococcal polysaccharide vaccine

^a^ Versus vaccine serotype

In the decision analysis model (Fig. 1), when considering public health outcomes, influenza outcomes included illness requiring no treatment, outpatient treatment, hospitalization, or death, based on probabilities from the literature [12]. Outcomes for pneumococcal disease included IPD and NBP, with risks of disability and mortality for each. NBP cases could either be treated as outpatient or inpatient, while all IPD cases were assumed to be hospitalized [13]. Individuals could have mild, moderate, or severe pertussis; with severe pertussis, there were additional risks of mortality or encephalopathy [14, 15]. Diphtheria and tetanus outcomes were not included in the analysis due to rarity of those diseases. Model outputs for vaccine-preventable illness, hospitalization, and death were included as proportions of the cohort, which are presented as percentages and as likelihoods per 100,000 in the cohort.

In the cost effectiveness analysis, vaccine-preventable illnesses occurring over the 10-year model time horizon had effectiveness modeled as lifetime per-person losses in quality adjusted life years (QALY) due to those illnesses; disability and death resulted in QALY losses based on the discounted life expectancy of the cohort. Costs of the 4 Pillars Program were calculated based on questionnaire data completed by study sites on personnel time and materials costs devoted to the program during its implementation and maintenance phases, which totaled $1.78 per eligible patient. These costs, which included estimates of time required for all components of the 4 Pillars, including physician immunization champions, were varied over ranges based on differences in observed site-specific costs and on alternative costing assumptions. These costs varied from $1700–$5400 per center per year, and are further enumerated in Additional file 1: Table S1. All other costs and quality of life utilities were obtained from the medical literature and U.S. databases [11, 12, 14, 16–18]. All costs were inflated as necessary to 2015 U.S. dollars using the Consumer Price Index. In accordance with recently updated recommendations for cost-effectiveness analysis [19], our results include both third party payer and societal perspectives. Third party payer perspective costs include direct medical costs from illness outcomes, vaccinations and intervention program, and cost of death; whereas societal perspective costs additionally included the costs of lost productivity from illness, disability, and death. Cost-effectiveness results were calculated by comparing each strategy’s per person total costs for the intervention, vaccination, and illness and effectiveness in quality adjusted life years to determine the incremental cost and incremental effectiveness between strategies. Dividing incremental cost by incremental effectiveness calculates the incremental cost effectiveness ratio (ICER), producing the incremental cost per quality adjusted life year gained for one strategy compared to the other.

## Results

The model demonstrated substantial public health benefits of the intervention over a 10-year period for adults 18–64 years with high-risk medical conditions (Table 2). Specifically, the intervention program reduced influenza cases by 1.37% and influenza hospitalizations by 0.028%. With the intervention program, the model also predicted smaller but substantial decreases in pertussis and in pneumococcal disease.

**Table 2:** Public health outcome predictions – vaccination programs in high-risk adults aged 18–64 years

	Strategy (cases per 100,000)	
	4 Pillars Program	No Program
Influenza		
Cases	32,898	34,270
Hospitalizations	679	707
Deaths	44	46
Pertussis		
Cases	1759	1815
Severe Cases	53	54
Deaths	0.454	0.468
Pneumococcal Disease		
IPD cases	162	168
IPD deaths	26	27
NBP hospitalized	1898	1926
NBP outpatient	17,226	17,479
NBP deaths	119	121

*IPD* Invasive pneumococcal disease, *NBP* Nonbacteremic pneumococcal pneumonia

Table 3 outlines cost-effectiveness analysis results from the model. From a third party payer perspective, considering only direct medical costs, the 4 Pillars Program per-person total vaccination and illness costs were $1642, $17.88 greater than no intervention, while losing 0.00063 QALYs compared with no program, thus the program had an ICER of $28,301 per QALY gained. From a societal perspective, adding lost productivity costs, the 4 Pillars Program had per-person total costs that were $31.15 less than no program while remaining more effective, thus dominating the no program strategy.

**Table 3:** Cost-effectiveness analysis of 4 Pillars Transformation Program in high-risk adults aged 18–64 years

Strategy	Cost per person	Incremental Cost	Effectiveness (QALY)	Incremental Effectiveness (QALY)	ICER^a^ ($/QALY)
3rd Party Payer Perspective					
No Program	$1624.44	–	−0.02808		
4 Pillars	$1642.32	$17.88	−0.02744	0.00063	$28,301
Societal Perspective					
4 Pillars	$3781.77	–	− 0.02744		
No Program	$3812.92	$31.15	−0.02808	−0.00063	Dominated^b^

^a^ICER = Incremental Cost-Effectiveness Ratio

^b^Costs more and less effective when compared to alternate strategy

When varying each parameter individually in 1-way sensitivity analysis over the ranges shown in Table 1, the intervention program remained favored from both third party and societal perspectives with each individual parameter variation when using a $100,000/QALY threshold, a commonly cited U.S. benchmark [20]. If the program was completely ineffective at increasing influenza vaccination rates or if influenza vaccine effectiveness was 20% yearly throughout the 10-year time horizon (base case 59%), then the program cost about $75,000 per QALY gained from a third-party payer perspective; these 2 parameters, both related to influenza vaccination, were the most sensitive to variation. With individual variation of all other parameters over clinically plausible ranges as listed in Table 1, model results were robust, with the intervention strategy remaining favored throughout.

Probabilistic sensitivity analysis results, where all parameters were simultaneously varied over distributions, are depicted in Fig. 2 as acceptability curves. At a $100,000/QALY gained threshold, the 4 Pillars Program was favored in 83.3% of the model iterations from a third party perspective and in 96.2% of model iterations from a societal perspective. From a societal perspective, the program was cost saving and more effective than no program in 75.7% of model iterations.

## Discussion

Our analysis suggests that implementing an intervention program, such as the 4 Pillars Program, to increase vaccination uptake prevented more illness and was likely economically favorable in an 18–64 year old adult population with immunocompromising conditions or other comorbid conditions that confer a high risk of vaccine-preventable disease. From a third-party payer perspective considering direct medical costs, the incremental cost-effectiveness ratio of $28,301/QALY gained with program use was well within the benchmark $100,000/QALY gained threshold for economic favorability; [20] from a societal perspective that additionally considered lost productivity costs, the intervention program was less costly and more effective than no intervention; i.e., a potentially cost-saving intervention that improves health outcomes. Results were robust in sensitivity analyses, with no individual parameter variation causing the intervention to cost more than $100,000 per QALY gained and simultaneous variation of all parameters showing that the intervention was highly likely to be favored over no intervention.

Currently, the proportion of adults with high-risk conditions in the U.S. ranges from 12.4% in the 19–24 year-old age group to 30.6% in persons 50–64 years of age [1]. These proportions are expected to grow as obesity rates continue to climb, with their attendant increases in diabetes, cardiovascular disease, and other obesity-related diseases. A practice-based intervention, such as the 4 Pillars Practice Transformation Program, is an excellent method to reach high-risk groups, because their chronic conditions typically increase the frequency with which they receive medical care. The modeled public health impact of the intervention program is notable, with substantial reductions in influenza cases and hospitalizations and in pneumococcal illness. This combination of public health benefit and economical reasonableness suggest that quality improvement efforts to increase high-risk adult vaccinations using the 4 Pillars Program are a worthwhile investment.

Many cost-effectiveness analyses of vaccine strategies can be found in the literature, mostly highly favorable toward vaccination, but fewer consider the cost-effectiveness of intervention programs designed to improve vaccination rates. Analyses examining the cost-effectiveness of vaccine uptake intervention programs mainly focused on adults aged 65 and older [9, 21–23]. One study compared the same 4 Pillars intervention in this analysis while the others examined different hypothetical programs of varying intensity in an elderly, minority population. These decision analysis-based studies found an increase in incremental effectiveness and slightly higher incremental costs when compared to no intervention, resulting in ICERs well below a $50,000/QALY gained threshold, indicating economic favorability.

### Strengths and limitations

This study is based on clinical trial results and incorporates both third-party and societal perspectives per recent guidelines [19]. All decision analyses and their results are subject to the parameters selected, although results were robust in 1-way and probabilistic sensitivity analyses. Influenza vaccination uptake and effectiveness were most sensitive to variation; thus, factors that interfere with program-related improvements in influenza vaccine uptake or diminish influenza vaccine effectiveness could impact the cost-effectiveness of the intervention. However, model results were not substantially affected by individual variation of all other parameter values, with the intervention remaining the favored strategy with individual and collective parameter variation. Another limitation is that we modeled the probability of receiving one vaccine as independent of receipt of other vaccines, in keeping with an overall public health impact perspective. In addition, we cannot separate the impact of each of the 4 Pillars. While the 4 Pillars program does allow practices to implement only those components of the program that each practice feels will be implementable and successful, studies have shown that implementation of only one or a relative few of the those components will not lead to success [24–26]. Finally, we assume that the pneumococcal polysaccharide vaccine is ineffective in preventing pneumococcal non-bacteremic pneumonia, which could be a controversial contention [27]. However, if this vaccine is effective against NBP, the intervention becomes even more favorable compared to no program.

## Conclusion

The 4 Pillars Practice Transformation Program is a cost-effective or cost-saving strategy, depending on the perspective taken, for averting vaccine preventable diseases in adults aged less than 65 years with medical conditions that place them at higher risk for influenza complications.

## Additional file

Additional file 1: Table S1. Description of data: Summary of intervention cost by center. (DOCX 17 kb)

## Abbreviations

CDC: Centers for Disease Control and Prevention
HIV: human immunodeficiency virus
ICER: Incremental cost effectiveness ratio
IPD: Invasive pneumococcal disease
NBP: Non-bacteremic pneumococcal pneumonia
PCV13: 13-valent pneumococcal conjugate vaccine
PPSV: 23-valent pneumococcal polysaccharide vaccine
QALY: Quality- adjusted life year
Tdap: Tetanus-diphtheria-acellular pertussis vaccine
U.S.: United States
