# Supplementary material for: Cost-effectiveness of increasing vaccination in high-risk adults aged 18–64 Years: a model-based decision analysis
Source: BMC Infect Dis. 2018 Jan 25;18:52. doi: 10.1186/s12879-018-2967-2 (PMC5785845; doi:10.1186/s12879-018-2967-2)
Supplement: Additional file 1: Table S1. — Description of data: Summary of intervention cost by center. (DOCX 17 kb) [file 12879_2018_2967_MOESM1_ESM.docx]

| \|  \|  \|  \|  \|  \|  \|  \|  \|  \| \| --- \| --- \| --- \| --- \| --- \| --- \| --- \| --- \| --- \|   **Table S1 – Cost summary - all centers, all years, average per center per year**   \|  \| \| \|  \|  \|  \|  \| \|  \| \|  \|  \| \| --- \| --- \| --- \| --- \| --- \| --- \| --- \| --- \| --- \| --- \| --- \| --- \| \| ***Questionnaire summary*** \| \| \|  \|  \|  \| ***Cost summary*** \| \|  \| \|  \|  \| \|  \| \| \|  \|  \|  \|  \| \|  \| \|  \|  \| \|  \| ***Item answered*** \| \| \|  \|  \| ***Costs - Assumption 1**** \| \| ***Costs - Assumption 2*†** \| \|  \|  \| \| ***Training*** \| **Yes** \| **No (or missing)** \| \| **Time (if yes) avg hours** \| **Hourly wage** \| ***Training*** \| **% of total** \| ***Training*** \| **%** \|  \|  \| \| Office administrator \| 4 \| 23 \| \| 1.83 \| $33.33 \| $61.11 \| 6.4% \| $9.05 \| 4.5% \|  \|  \| \| Non clinical staff \| 3 \| 24 \| \| 2.50 \| $13.50 \| $33.75 \| 3.5% \| $3.75 \| 1.8% \|  \|  \| \| Physicians \| 6 \| 21 \| \| 4.45 \| $166.67 \| $741.13 \| 77.3% \| $164.69 \| 81.1% \|  \|  \| \| Nurse \| 6 \| 21 \| \| 3.62 \| $30.00 \| $108.67 \| 11.3% \| $24.15 \| 11.9% \|  \|  \| \| Med assistant \| 3 \| 24 \| \| 0.92 \| $15.00 \| $13.75 \| 1.4% \| $1.53 \| 0.8% \|  \|  \| \|  \|  \|  \| \|  \|  \| **$958.40** \|  \| **$203.17** \|  \| ***Training total*** \| \|  \| \|  \|  \|  \| \|  \|  \|  \|  \|  \|  \|  \| \|  \| \| ***Implementation*** \|  \|  \| \|  \|  \| ***Implementation*** \| **% of total** \| ***Implementation*** \| **%** \|  \| \|  \| \| Office administrator \| 12 \| 15 \| \| 3.51 \| $33.33 \| $116.88 \| 3.0% \| $51.95 \| 2.8% \|  \| \|  \| \| Physicians \| 14 \| 13 \| \| 17.45 \| $166.67 \| $2,907.59 \| 74.1% \| $1,507.64 \| 80.5% \|  \| \|  \| \| Nurses \| 13 \| 14 \| \| 11.88 \| $30.00 \| $356.50 \| 9.1% \| $171.65 \| 9.2% \|  \| \|  \| \| Med assistant \| 7 \| 20 \| \| 31.31 \| $15.00 \| $469.61 \| 12.0% \| $121.75 \| 6.5% \|  \| \|  \| \| Supplies \| 7 \| 20 \| \| - \| - \| $74.83 \| 1.9% \| $19.40 \| 1.0% \|  \| \|  \| \|  \|  \|  \| \|  \|  \| **$3,925.42** \|  \| **$1,872.39** \|  \| ***Implementation total*** \| \|  \| \|  \|  \|  \| \|  \|  \|  \|  \|  \|  \|  \| \|  \| \|  \|  \|  \| \|  \|  \| **$4,883.81** \|  \| **$2,075.56** \|  \| ***Total annual cost*** \| \|  \| \|  \|  \|  \| \|  \|  \|  \|  \|  \|  \|  \| \|  \| | | | | | | | | | | |  |  |  |  |  |  |
| --- | --- | --- | --- | --- | --- | --- | --- | --- | --- | --- | --- | --- | --- | --- | --- | --- | --- | --- | --- | --- | --- | --- | --- | --- | --- | --- | --- | --- | --- | --- | --- | --- | --- | --- | --- | --- | --- | --- | --- | --- | --- | --- | --- | --- | --- | --- | --- | --- | --- | --- | --- | --- | --- | --- | --- | --- | --- | --- | --- | --- | --- | --- | --- | --- | --- | --- | --- | --- | --- | --- | --- | --- | --- | --- | --- | --- | --- | --- | --- | --- | --- | --- | --- | --- | --- | --- | --- | --- | --- | --- | --- | --- | --- | --- | --- | --- | --- | --- | --- | --- | --- | --- | --- | --- | --- | --- | --- | --- | --- | --- | --- | --- | --- | --- | --- | --- | --- | --- | --- | --- | --- | --- | --- | --- | --- | --- | --- | --- | --- | --- | --- | --- | --- | --- | --- | --- | --- | --- | --- | --- | --- | --- | --- | --- | --- | --- | --- | --- | --- | --- | --- | --- | --- | --- | --- | --- | --- | --- | --- | --- | --- | --- | --- | --- | --- | --- | --- | --- | --- | --- | --- | --- | --- | --- | --- | --- | --- | --- | --- | --- | --- | --- | --- | --- | --- | --- | --- | --- | --- | --- | --- | --- | --- | --- | --- | --- | --- | --- | --- | --- | --- | --- | --- | --- | --- | --- | --- | --- | --- | --- | --- | --- | --- | --- | --- | --- | --- | --- | --- | --- | --- | --- | --- | --- | --- | --- | --- | --- | --- | --- | --- | --- | --- | --- | --- | --- | --- | --- | --- | --- | --- | --- | --- | --- | --- | --- | --- | --- | --- | --- | --- | --- | --- | --- | --- | --- | --- | --- | --- | --- | --- | --- | --- | --- | --- | --- | --- | --- | --- | --- | --- | --- | --- | --- | --- | --- | --- | --- | --- | --- | --- | --- | --- | --- | --- | --- | --- | --- | --- | --- | --- | --- | --- | --- | --- | --- | --- | --- | --- | --- | --- |
|  | | | | | | | | | | | |  | |  |  |  |
| * Assumes "no or missing" answers are oversights and that the average for all centers is the same as the average of those answering "yes" | | | | | | | | | | | |  | |  |  |  |
|  |  |  |  | |  | |  | | | | |  | |  |  |  |
| † Assumes that "no or missing" means no costs and calculates average based on that assumption | | | | | | | | | | |  |  |  |  |  |  |
|  |  |  |  | |  | |  | |  | | |  |  |  |  |  |
|  |  |  |  | |  | |  | |  | | |  |  |  |  |  |
|  |  |  |  | |  | |  | |  | | |  |  |  |  |  |
|  |  |  |  | |  | |  | |  | | |  |  |  |  |  |
|  |  |  |  | |  | |  | |  | | |  |  |  |  |  |
|  |  |  |  | |  | |  | |  | | |  |  |  | |  |
|  |  |  |  | |  | |  | | | |  |  |  |  |  |  |
|  |  |  |  | |  | |  | | |  | |  |  |  |  |  |
|  |  |  |  | |  | |  | | |  | |  |  |  |  |  |
|  |  |  |  | |  | |  | | |  | |  |  |  |  |  |
|  |  |  |  | |  | |  | | |  | |  |  |  |  |  |
|  |  |  |  | |  | |  | | |  | |  |  |  |  |  |
|  |  |  |  | |  | |  | | |  | |  |  |  |  |  |
|  |  |  |  | |  | |  | | |  | |  |  |  | | |
|  |  |  |  | |  | |  | | |  | |  |  |  |  |  |
|  |  |  |  | |  | |  | | | |  |  |  |  | |  |
|  |  |  |  | |  | |  | | | |  |  |  |  |  |  |
|  | | | | | | | | | | | | |  |  |  |  |
|  |  |  | |  | |  | |  | | |  |  |  |  |  |  |
|  | | | | | | | | | | |  |  |  |  |  |  |
